# Causal Associations of PM2.5 and GDM: A Two-Sample Mendelian Randomization Study

**DOI:** 10.3390/toxics11020171

**Published:** 2023-02-13

**Authors:** Yi Yang, Xianli Ma, Weiyi Pang, Caina Jiang

**Affiliations:** 1 Guangxi Engineering Research Center for Pharmaceutical Molecular Screening and Druggability Evaluation, Guilin Medical University, Guilin 541199, China; 2 School of Pharmacy, Guilin Medical University, Guilin 541199, China; 3 Guangxi Key Laboratory of Environmental Exposomics and Entire Lifecycle Heath, Guilin Medical University, Guilin 541199, China

**Keywords:** air pollution, gestational diabetes mellitus, MR, causality, risk factor

## Abstract

Epidemiological studies have linked particulate matter (PM2.5) to gestational diabetes mellitus (GDM). However, the causality of this association has not been established; Mendelian randomization was carried out using summary data from genome-wide association studies (GWAS). For the analysis of the causal relationship between PM2.5 and GDM, the inverse variance weighted (IVW) method was used. The exposure data came from a GWAS dataset of IEU analysis of the United Kingdom Biobank phenotypes consisting of 423,796 European participants. The FinnGen consortium provided the GDM data, which included 6033 cases and 123,000 controls. We also performed multivariate MR (MVMR), adjusting for body mass index (BMI) and smoking. As a result, we found that each standard deviation increase in PM2.5 is associated with a 73.6% increase in the risk of GDM (OR: 1.736; 95%CI: 1.226–2.457). Multivariable MR analysis showed that the effect of PM2.5 on GDM remained after accounting for BMI and smoking. Our results demonstrate a causal relationship between PM2.5 and GDM.

## 1. Introduction

Gestational diabetes mellitus (GDM) is defined by glucose intolerance during pregnancy, resulting in different levels of hyperglycemia. Over the last few decades, the occurrence of GDM has seen a stable increase [1,2,3]. As per the International Diabetes Federation (IDF), the global pooled prevalence of GDM in pregnant women in 2021 was 14.0% [4]. GDM has developed into a significant public health problem. There are well-known risk factors for GDM, including obesity, a high maternal age, and a family history of diabetes [5,6]. These, however, might not fully explain the seasonal variations in the prevalence of GDM [7]. Therefore, we targeted our attention on lifestyle or environment-related risk factors, such as PM2.5.

Particulate matter 2.5 (PM2.5) is a significant air contaminant that seriously threatens human health [8]. PM2.5 has been linked to various health issues, including respiratory, cardiovascular, and cerebrovascular disease [9]. Currently, epidemiological study regarding PM2.5 and GDM is controversial [10]. Several studies in the past have shown a link between PM2.5 and GDM [11,12]. However, another meta-analysis of cohort studies has revealed an absence of association [13]. This may be related to bias due to the small sample size, inadequate follow-up, and many unconfounded factors [14]. Therefore, the causal relationship between PM2.5 and GDM is unclear, and we require more robust evidence to support it.

The results of previous research have shown that PM2.5 exposure leads to adverse health effects influenced by changes in gene expression [15]. Elderly participants with PARP4 G-C-G and ERCC1 T-C haplotypes were susceptible to elevated fasting glucose levels under the influence of PM2.5 exposure [16]. The incidence of lower respiratory tract infections was significantly increased in infants with the STP1 (rs1695) AG or GG or Nrf2 (rs6726395) GG genotypes under prenatal indoor PM2.5 exposure [17]. However, no studies have analyzed the effect of genetic polymorphisms and PM2.5 on gestational diabetes. Mendelian randomization (MR) uses genetic variants closely related to exposure as internal instrumental variables (IVs) to explore causal relationships between exposure and outcome [18]. Because gametes follow Mendel’s laws of inheritance in their formation, the random allocation of the alleles at conception eliminates confounding bias and adheres to the temporality of causality [19,20]. Consequently, a two-sample MR analysis was performed to determine the causal link between PM2.5 and GDM.

## 2. Materials and Methods

### 2.1. Study Design

We conducted a two-sample MR analysis to identify the causal associations between PM2.5 and GDM using publicly available summary datasets from two genome-wide association studies (GWAS) [21]. Previous observational studies have shown that BMI and smoking are important risk factors for the development of GDM. Therefore, we further performed multivariable Mendelian randomization (MVMR) analyses to estimate the direct causal effect of PM2.5 on the risk of GDM.

### 2.2. Data Sources

For the PM2.5 exposure dataset, the summary genetic data on PM2.5 were obtained from the UK Biobank GWAS, which included 423,796 European participants. The study was based on the ESCAPE project (European Study of Cohorts for Air Pollution Effects), which used the LUR model to estimate PM2.5 pollution concentrations at the home addresses of study participants [22]. The mean (±standard deviation) PM2.5 level was 9.99 (±1.06) µg/m^3^ in the GWAS. The dataset was publicly available from the MRC IEU OpenGWAS data and MR-Base with the GWAS-ID ukb-b-10817. It was the output of the GWAS pipeline using the Phesant-derived variables from the UK Biobank.

Data on genetic variants associated with GDM were obtained from the FinnGen consortium, and an ongoing Finnish national study started in 2017. The dataset of GDM with the GWAS-ID of finn-b-O15_PREG_DM was downloaded from FinnGen, which included 6,033 GDM cases in 123,000 women, and the dataset consisted entirely of Europeans [23]. 

In addition, we obtained summary data on BMI and smoking from the GWAS. The summary data for BMI came from the GIANT consortium. These data were obtained from a meta-analysis of up to 339,224 European individuals from 125 studies [24]. As for the smoking GWAS, there were 88,601 cases and 201,126 controls from the Neale Lab consortium. Both GWAS data were from populations of European origin, and their summary information is presented in Supplementary Table S1.

### 2.3. Genetic Variants

In the MR analysis, the single nucleotide polymorphisms (SNPs) were screened from the exposure dataset as the IVs [25]. It is necessary that IVs meet three assumptions: (1) IVs must be related to PM2.5; (2) IVs should be independent of confounding factors; and (3) IVs are not directly associated with GDM [26]. 

To fulfill the three assumptions of the MR analysis, we applied the following method to select the IVs. It should be noted that we chose *p* < 5 × 10^−8^ as the general genome-wide significance threshold. However, if *p* < 5 × 10^−8^ is used as a screening criterion, only eight SNPs in this database meet it. It has been shown that after the linear regression of each genetic variant on risk factors at *p* < 1 × 10^−5^ as a screening criterion, the results showed the low possibility of weak instrumental variable bias in MR analysis. Therefore, we chose SNPs as IVs associated at this significance level since there were not enough SNPs associated at the genome-wide significant threshold of 5 × 10^−8^ [27,28]. Secondly, we used the PhenoScanner tool to ensure whether the IVs were significantly correlated with the risk factors for GDM [29,30]. In addition, we used the ’’clump_data’’ function on MR-Base to select independent SNPs (linkage disequilibrium (LD) R^2^ = 0.001, >10,000 kb) [31]. If no SNP associated with PM2.5 was found in the GDM database, then proxy SNPs were searched for with a minimum LD R^2^ = 0.8 [32]. Palindrome SNPs were retained based on the criterion that the MAF < 0.3 [33].

We obtained relevant data from two datasets, including SNP sites, the effect allele (EA), the non-effect allele (non-EA), the minor allele frequency (MAF), the beta coefficient (BETA), the standard error (SE), and the *p*-value.

### 2.4. Statistical Analysis 

For the evaluation of the causal link between PM2.5 and GDM, the inverse variance weighted (IVW) method was used. We supplemented our verification using MR–Egger regression, weighted median, weighted mode, and simple mode to enhance accuracy and stability [34]. We used MR–Egger regression to test whether pleiotropy in IVs was present and whether it impacted the results. It was judged that there was no effect of pleiotropy in IVs if the MR–Egger intercept was close to 0 or *p* > 0.05 [35]. For the IVW method, Cochran’s Q test was applied to examine heterogeneity between IVs [36]. The result of *p* > 0.05 indicated that there was no heterogeneity. To remove random errors from screening IVs, we used a leave-one-out sensitivity test, eliminating each SNP individually, to determine whether our results were influenced by a particular SNP [37]. Finally, the F statistic was calculated to determine whether the screened IVs had a weak instrumental variable bias. If F was >10, there was no weak instrumental bias [38].

All analyses were conducted with the TwoSampleMR package and R Foundation version 4.2.0. The statistical significance was set at *p* < 0.05.

## 3. Results

We used *p* < 1 × 10^−5^ as a screening criterion for SNPs with large P values in multiple chain imbalances (r^2^ > 0.001) and obtained 99 IVs with LD (r^2^ < 0.001) based on the PM2.5 dataset. In this case, we removed one SNP (rs7093269), a palindromic SNP with an intermediate allele frequency. The PhenoScanner tool was used to verify that, in the instrumental variables we identified, 13 SNPs were associated with the possible mechanistic pathways (including BMI, smoking, a family history of diabetes, etc.) of GDM (Supplementary Table S2). In the end, 85 SNPs were identified for further MR analysis (Supplementary Table S3).

**Figure 1:**
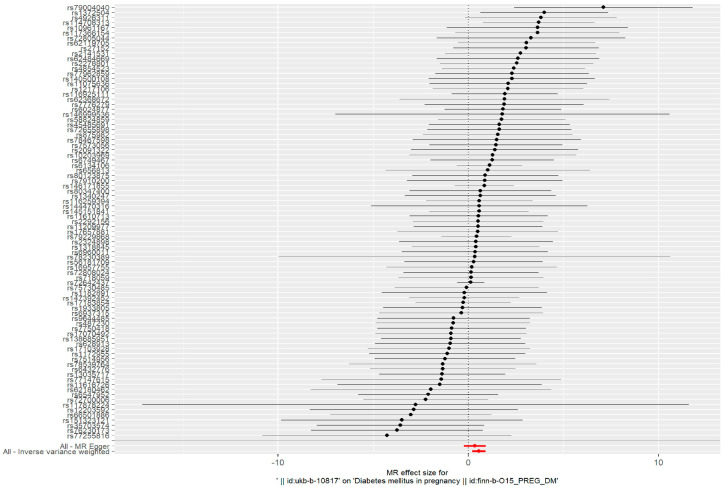
Forest plots of the causal effects between PM2.5-associated SNPs and risk of GDM.

The IVW method was used as the primary analysis to evaluate the association between PM2.5 and GDM risk. The results of Cochran’s Q test indicated no heterogeneity (*p* = 0.859). The IVW method showed an association between PM2.5 and GDM risk (odds ratio (OR) = 1.736, 95% confidence interval (95%CI) = (1.226–2.457), *p* = 0.002) (Figure 1).

**Figure 2:**
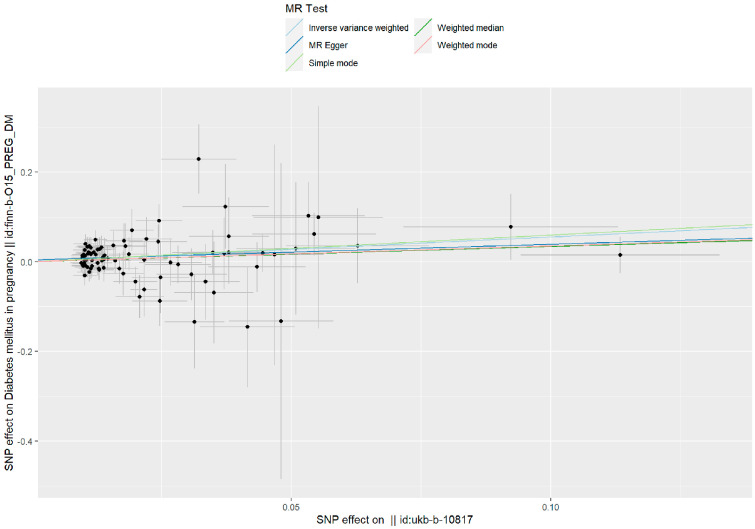
A scatter plot is used to visualize the causal effect between PM2.5 and GDM. As shown by the scatter plots of the inverse-variance weighted (IVW) method, the MR–Egger regression method, the weighted median, the weighted mode, and the simple mode, the slope of the straight line indicates the magnitude of the causal association.

**Figure 3:**
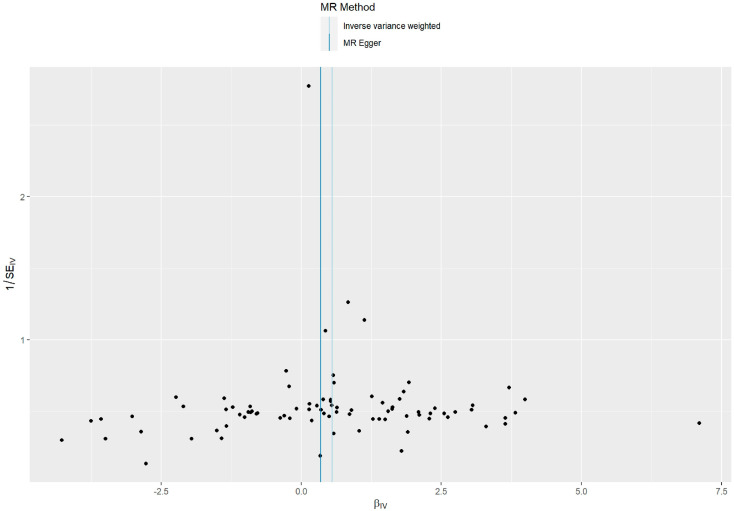
A funnel plot of the relationship between the causal effect of PM2.5 on GDM. Funnel plots showed the relationship between the causal effect of PM2.5 on GDM estimated with each SNP as a separate instrument and the inverse of the standard error of the causal estimate. Each black point represents a valid instrumental SNP. The vertical line represents the estimated causal effect of all instrumental SNPs using the two different methods.

**Figure 4:**
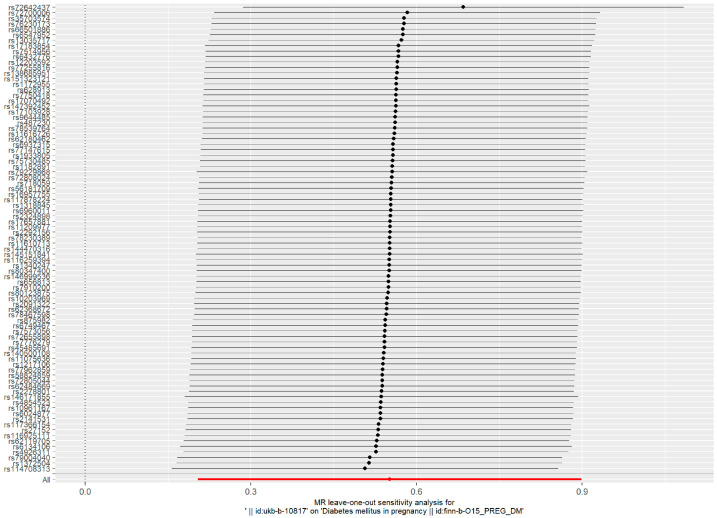
Forest plot of the ’’leave-one-out’’ sensitivity analysis method to show the influence of individual SNPs on the results. The red point indicates the IVW estimates using all SNPs.

Such associations were consistent, although non-significant, in MR–Egger, weighted median, weighted model, and simple model (Table 1 and Figure 2). MR–Egger demonstrated no evidence of horizontal pleiotropy (*p* = 0.395). The funnel plot (Figure 3) was symmetrical, indicating that our analysis did not influence pleiotropy. A leave-one-out analysis was used to analyze the IVW results (Figure 4). We deleted each SNP individually and obtained *p* < 0.05, which was consistent with the results of the IVW method in the analysis of the causal effects, indicating that no non-specific SNPs could have influenced the causal estimation results. Finally, the F statistic showed a range of 19.56–69.92 for each SNP, excluding the weak instrumental variable bias. Using the mRnd method, we calculated the phenotypic variance explained to be 5.85%. The OR was 1.736 when the estimated statistical power was 100% with the current sample size. We used IVW as the primary criterion for causality because of the absence of horizontal pleiotropy in the analysis. We considered PM2.5 as a risk factor for the incidence of GDM. 

Multivariate results showed that the association between PM2.5 and GDM risk remained statistically significant after adjusting for BMI, smokng, and all other factors (Supplementary Table S4).

## 4. Discussion

We performed an MR analysis to test the causality of PM2.5 on GDM. Our results showed that the risk of GDM increased by 73.6% (OR: 1.736; 95%CI: 1.226–2.457) for each standard deviation increase in PM2.5 by using a cutoff value of *p* < 1 × 10^−5^ to select the instrumental variable. In addition, the results of the MR–Egger, weighted median, weighted model, and simple model tests were not significant. This result may be caused by residual pleiotropy. Further MVMR analysis showed that the effect of PM2.5 on GDM remained after taking into account risk factors such as smoking, BMI, and a family history of diabetes.

GDM and PM2.5 have been linked in previous observational studies, but their results have been controversial [39,40]. A Florida study found an increased risk of GDM among pregnant women exposed to PM2.5 (OR: 1.20, 95%CI, 1.13–12.26) [12]. Similarly, a Rhode Island study found higher PM2.5 levels were associated with higher chances of developing GDM during the second trimester (OR: 1.08, 95%CI: 1.00–1.15) [41]. In a study from New York City, the odds of GDM were higher among those exposed to PM2.5 in the second trimester (OR: 1.06, 95%CI: 1.02–1.10) [42]. However, one cohort study in Massachusetts found no association between PM2.5 and GDM [43]. Even in a case–control study in California, PM2.5 has shown a negative association with GDM [10]. These controversies may be related to small patient samples, confounding factors, and different study designs.

Currently, it is not clear through which biological mechanisms PM2.5 increases the risk of GDM. Because of its small size, PM2.5 can reach various organs through the blood circulation, including the pancreas, leading to adverse health effects. The study found that PM2.5 exposure increased reactive oxygen species (ROS) levels [44], and that the accumulation of ROS increases oxidative stress, leading to β-cell dysfunction [45]. PM2.5 exposure also induces insulin resistance through inflammation, disrupting the insulin receptor signaling pathway [11]. In addition, an animal study showed that pancreatic glutathione peroxidase (GSH-Px) was significantly decreased, methane dicarboxylic aldehyde (MDA) was increased, and inflammation was observed around the pancreas; additionally, pancreatic GLUT2 expression was decreased in rats after PM2.5 exposure [46]. This provides evidence that PM2.5 exposure leads to pancreatic damage and glycemic consequences through oxidative responses and inflammation. 

Our study has the following crucial strengths: to our knowledge, this is the first time PM2.5 and GDM have been analyzed causally using two-sample MR. Previous epidemiologic studies have suggested a controversial relationship between PM2.5 and GDM, which may be influenced by confounding factors and reverse causality. Based on MR analysis, Mendel’s law of independent assignment chose genetic variation as the exposure factor, making the findings more reliable. Secondly, the genes appear before the disease is present, which excludes the effect of reverse causality. In addition, MR analysis was conducted in conjunction with data from published GWAS pooled studies, and the large sample size could improve the efficacy of the test. Our results provide a new theoretical and experimental basis for preventing population health risks from air pollutants.

There were several limitations to our study. To begin with, the GWAS datasets included in the MR analysis were from Europe, and further studies are needed to be conducted on populations of other countries to improve the generalizability of the results. Additionally, we used the PhenoScanner tool to meet the assumptions of the MR analysis: IVs are not directly associated with GDM. However, certain unpublished SNPs may be linked to GDM and thus affect our findings. Our results were obtained based on a significance level of 1 × 10^−5^. Although there were not enough SNPs associated with the genome-wide significance threshold of 5 × 10^−8^, we also performed a two-sample MR analysis. The trend of increased PM2.5 concentrations with an elevated risk of GDM was consistent with our results, although it was not statistically significant. We will seek further evidence through more extensive studies.

## 5. Conclusions

In conclusion, our results imply a possible causal relationship between PM2.5 and GDM. Additional experimental and mechanistic studies are required to verify the validity of the findings presented in this study.

## Figures and Tables

**Table 1:** Two-sample Mendelian randomization for PM2.5 on GDM risk.

Method	N SNPs	Beta Coefficient	SE	OR (95%CI)	*p*
IVW	85	0.551	0.177	1.736 (1.226–2.457)	0.002
MR–Egger	85	0.350	0.295	1.418 (0.795–2.530)	0.240
Weighted median	85	0.339	0.298	1.404 (0.782–2.519	0.256
Weighted mode	85	0.351	0.292	1.421 (0.802–2.519)	0.232
Simple mode	85	0.598	0.594	1.818 (0.567–5.829)	0.317

The number of single nucleotide polymorphisms, SNPs; SE, standard error; OR, odds ratio; CI, confidence interval.

## Data Availability

Publicly available datasets were analyzed in this study. These data can be found in the UK Biobank and FinnGen.

## Supplementary Materials

The following supporting information can be downloaded at: https://www.mdpi.com/article/10.3390/toxics11020171/s1, Table S1: A brief description of the genome-wide association study data used in this study; Table S2: Removed SNPs were associated with the possible mechanistic pathways of GDM; Table S3: Characteristics of SNPs used in the MR analysis in the summary statistics reported in the GWAS on PM2.5 and GDM; Table S4: Causal estimates of PM2.5 on GDM in multivariable MR; STROBE-MR checklist: STROBE-MR checklist of Mendelian randomization studies. References [47,48] are cited in the supplementary materials.
