# Supplementary material for: Causal Associations of PM2.5 and GDM: A Two-Sample Mendelian Randomization Study
Source: Toxics. 2023 Feb 13;11(2):171. doi: 10.3390/toxics11020171 (PMC9961059; doi:10.3390/toxics11020171)
Supplement: Supplementary file 1 [file toxics-11-00171-s001.zip › STROBE-MR checklist.pdf]

STROBE-MR checklist of Mendelian randomization study

Item No.	Section	Checklist item	Page No.	Relevant text from manuscript
1	TITLE and ABSTRACT	Indicate Mendelian randomization (MR) as the study's design in the title and/or the abstract if that is a main purpose of the study	1	Causal Associations of PM2.5 and GDM : A Two-Sample Mendelian Randomization Study
	INTRODUCTION			
2	Background	Explain the scientific background and rationale for the reported study. What is the exposure? Is a potential causal relationship between exposure and outcome plausible? Justify why MR is a helpful method to address the study question	1,2	It is explained in the first two paragraphs of the Introduction the scientific background and rationale of the study. Next, we illustrated the importance of MR in the third paragraph.
3	Objectives	State specific objectives clearly, including pre-specified causal hypotheses (if any). State that MR is a method that, under specific assumptions, intends to estimate causal effects	2	We clearly describe the objectives in paragraph 3 in the Introduction.
	METHODS			
4	Study design and data sources	Present key elements of the study design early in the article. Consider including a table listing sources of data for all phases of the study. For each data source contributing to the analysis, describe the following:	2	We conducted a two-sample MR analysis to identify the causal associations between PM2.5 and GDM by using publicly available summary datasets from two genome-wide association studies (GWAS) .In Materials and Methods we specify the study design and data sources, respectively. Information from the GWAS database is added to Table1 in the supplementary material.
	a)	Setting: Describe the study design and the underlying population, if possible. Describe the setting, locations, and relevant dates, including periods of recruitment, exposure, follow-up, and data collection, when available.		
	b)	Participants: Give the eligibility criteria, and the sources and methods of selection of participants. Report the sample size, and whether any power or sample size calculations were carried out prior to the main analysis		No ethical statement is required for this study.

	c)	Describe measurement, quality control and selection of genetic variants		
	d)	For each exposure, outcome, and other relevant variables, describe methods of assessment and diagnostic criteria for diseases		
	e)	Provide details of ethics committee approval and participant informed consent, if relevant		
5	Assumptions	Explicitly state the three core IV assumptions for the main analysis (relevance, independence and exclusion restriction) as well assumptions for any additional or sensitivity analysis	2	Related information is presented in Materials and Methods.<br>It is necessary that IV meets three assumptions: (1) IVs must be related to PM2.5; (2) IVs should be independent of confounding factors; (3) IVs are not directly associated with GDM.
6	Statistical methods: main analysis	Describe statistical methods and statistics used	2,3	All main statistical methods are reported in the "Statistical Analysis" and "Genetic variants" section. For the evaluation of the causal link between PM2.5 and GDM, the inverse variance weighted (IVW) method was used. We supplemented our verification by using MR-Egger regression, weighted median, weighted mode, and simple mode to enhance accuracy and stability.<br>Also, we further performed separate MVMR analyses to estimate the direct causal effect of PM2.5 on the risk of GDM.
	a)	Describe how quantitative variables were handled in the analyses (i.e., scale, units, model)		
	b)	Describe how genetic variants were handled in the analyses and, if applicable, how their weights were selected		
	c)	Describe the MR estimator (e.g. two-stage least squares, Wald ratio) and related statistics. Detail the included covariates and, in case of two-sample MR, whether the same covariate set was used for adjustment in the two samples		
	d)	Explain how missing data were addressed		
	e)	If applicable, indicate how multiple testing was addressed		
7	Assessment of assumptions	Describe any methods or prior knowledge used to assess the assumptions or justify their validity	3	We selected variants that were associated with the exposures at GWAS significance

				i.e. P< 1×10^{-5} . The PhenoScanner tool was used to ensure whether the IVs were significantly correlated with the risk factors for GDM.<br>We further performed separate MVMR analyses to estimate the direct causal effect of PM2.5 on the risk of GDM.<br>The relevant information is all reported in the Materials and Methods.
8	Sensitivity analyses and additional analyses	Describe any sensitivity analyses or additional analyses performed (e.g. comparison of effect estimates from different approaches, independent replication, bias analytic techniques, validation of instruments, simulations)	3	All sensitivity analyses are reported under the “ Statistical Analysis” section under the Materials and Methods.
9	Software and pre-registration		3	
	a)	Name statistical software and package(s), including version and settings used		With the TwosampleMR package and R Foundation version 4.2.0, all analyses were conducted.
	b)	State whether the study protocol and details were pre-registered (as well as when and where)		No pre-registration.
	RESULTS			
10	Descriptive data		2,supplementary material	
	a)	Report the numbers of individuals at each stage of included studies and reasons for exclusion. Consider use of a flow diagram		The information about our dataset is given in Table S1.<br>For the PM2.5 exposure dataset, the summary genetic data on PM2.5 were obtained from the UK biobank GWAS and the dataset of GDM with the GWAS-ID of finn-b-O15_PREG_DM were downloaded from Finngen. Related information can be found in the UK biobank and FinnGen.
	b)	Report summary statistics for phenotypic exposure(s), outcome(s), and other relevant variables (e.g. means, SDs, proportions)		
	c)	If the data sources include meta-analyses of previous studies, provide the assessments of heterogeneity across these studies		
	d)	For two-sample MR:		

		i. Provide justification of the similarity of the genetic variant-exposure associations between the exposure and outcome samples<br>ii. Provide information on the number of individuals who overlap between the exposure and outcome studies		This manuscript was not included in the meta-analysis of previous studies.
11	Main results		3,4	
	a)	Report the associations between genetic variant and exposure, and between genetic variant and outcome, preferably on an interpretable scale		The associations between all IVs used in our analyses and our exposures and outcomes are reported in Table1, S2, S3 and S4.
	b)	Report MR estimates of the relationship between exposure and outcome, and the measures of uncertainty from the MR analysis, on an interpretable scale, such as odds ratio or relative risk per SD difference		According to our limited conditions, we found that the risk of GDM increased by 73.6% (OR: 1.736; 95% CI: 1.226-2.457) for each standard deviation increase in PM2.5 by using a cutoff value of $p < 1 \times 10^{-5}$ to select the instrumental variable. (Firgue1-4).<br>Multivariate results showed that the association between PM2.5 and GDM risk remained statistically significant after adjusting for BMI, smoking, and all factors.
	c)	If relevant, consider translating estimates of relative risk into absolute risk for a meaningful time period		
	d)	Consider plots to visualize results (e.g. forest plot, scatterplot of associations between genetic variants and outcome versus between genetic variants and exposure)		
12	Assessment of assumptions		3,4	
	a)	Report the assessment of the validity of the assumptions		We supplemented our verification by using MR-Egger regression, weighted median, weighted mode, and simple mode to enhance accuracy and stability. To test if pleiotropy in IVs was present and whether it had an impact on the results, we used MR-Egger regression For the IVW method, the Cochran Q test was applied to examine heterogeneity between IVs. To remove random errors arising from screening IVs, we used a leave-one-out sensitivity test, eliminating each SNP individually, to determine whether our results were influenced by a particular SNP.
	b)	Report any additional statistics (e.g., assessments of heterogeneity across genetic variants, such as I2, Q statistic or E-value)		

				Finally, the F statistic was calculated to determine whether the screened IVs had weak instrumental variable bias.<br>All methods and results are described under “Statistical Analysis” and “Results”.
13	Sensitivity analyses and additional analyses		4,5	
	a)	Report any sensitivity analyses to assess the robustness of the main results to violations of the assumptions		Relevant information is given in the results section.
	b)	Report results from other sensitivity analyses or additional analyses		In addition we performed various MVMR models including adjustment for BMI, smoking, and all factors.
	c)	Report any assessment of direction of causal relationship (e.g., bidirectional MR)		A leave-one-out analysis was used for the analysis of the IVW results (Figure 4). We deleted each SNP individually and obtained $P < 0.05$, which is consistent with the results of the IVW method in the analysis of the causal effects, indicating that there were no non-specific SNPs that could have influenced the causal estimation results.
	d)	When relevant, report and compare with estimates from non-MR analyses		
	e)	Consider additional plots to visualize results (e.g., leave-one-out analyses)		
	DISCUSSION			
14	Key results	Summarize key results with reference to study objectives	5	We describe key results in the first paragraph of the discussion section.
15	Limitations	Discuss limitations of the study, taking into account the validity of the IV assumptions, other sources of potential bias, and imprecision. Discuss both direction and magnitude of any potential bias and any efforts to address them	6	The limitations are reported in paragraph 5 of our discussion.<br>To begin with, both GWAS datasets included in the MR analysis were from Europe, and further studies were needed to be conducted on populations of other countries for the generalizability of the results.

16	Interpretation		5,6	
	a)	Meaning: Give a cautious overall interpretation of results in the context of their limitations and in comparison with other studies		a) Reported in paragraphs 1 of the Discussion.<br>b) Discussion – paragraph 2-3<br>c) Discussion – paragraph 4
	b)	Mechanism: Discuss underlying biological mechanisms that could drive a potential causal relationship between the investigated exposure and the outcome, and whether the gene-environment equivalence assumption is reasonable. Use causal language carefully, clarifying that IV estimates may provide causal effects only under certain assumptions		
	c)	Clinical relevance: Discuss whether the results have clinical or public policy relevance, and to what extent they inform effect sizes of possible interventions		
17	Generalizability	Discuss the generalizability of the study results (a) to other populations, (b) across other exposure periods/timings, and (c) across other levels of exposure	5	We discuss potential caveats in terms of generalizability of results in paragraph 5.
	OTHER INFORMATION			
18	Funding	Describe sources of funding and the role of funders in the present study and, if applicable, sources of funding for the databases and original study or studies on which the present study is based	6	This work is supported by the Open Project Program of Guangxi Key Laboratory of Environmental Exposomics and Entire Lifecycle Health, Guilin Medical University (grant number 2022-GKLEH-08) and Guangxi Science and Technology Base and Talent Special Project (grant number AD18050005).<br>This study is based on a published database and does not require ethical approval.
19	Data and data sharing	Provide the data used to perform all analyses or report where and how the data can be accessed, and reference these sources in the article. Provide the statistical code needed to reproduce the results	6	This study is based on a published database and does not require ethical approval.

		in the article, or report whether the code is publicly accessible and if so, where		With the TwosampleMR package and R Foundation version 4.2.0, all analyses were conducted.
20	Conflicts of Interest	All authors should declare all potential conflicts of interest	6	The authors declare no conflict of interest.

This checklist is copyrighted by the Equator Network under the Creative Commons Attribution 3.0 Unported (CC BY 3.0) license.

1. Skrivankova VW, Richmond RC, Woolf BAR, Yarmolinsky J, Davies NM, Swanson SA, et al. Strengthening the Reporting of Observational Studies in Epidemiology using Mendelian Randomization (STROBE-MR) Statement. JAMA. 2021, 326(16):1614-1621.

2. Skrivankova VW, Richmond RC, Woolf BAR, Davies NM, Swanson SA, VanderWeele TJ, et al. Strengthening the Reporting of Observational Studies in Epidemiology using Mendelian Randomisation (STROBE-MR): Explanation and Elaboration. BMJ. 2021;375:n2233.
