# Supplementary material for: Causal Associations of PM2.5 and GDM: A Two-Sample Mendelian Randomization Study
Source: Toxics. 2023 Feb 13;11(2):171. doi: 10.3390/toxics11020171 (PMC9961059; doi:10.3390/toxics11020171)
Supplement: Supplementary file 1 [file toxics-11-00171-s001.zip › Supplementary materials.pdf]

Table S1 . Brief description of the genome-wide association study data used in this study.

Expourse/Outcome	GWAS-id	Population	ncase	Sample size	Number of SNPs
PM2.5	ukb-b-10817	European	NA	423,796	9,851,867
BMI	ieu-a-835	European	NA	322,154	2,554,668
Smoking	ukb-a-39	European	88,601	289,727	10,894,596
GDM	finn-b-O15_PREG_DM	European	6,033	123,000	16,379,684

BMI, body mass index; GDM, gestational diabetes mellitus; SNP, single nucleotide polymorphism.

Table S2 . Removed SNPs were associated with the possible mechanistic pathways of GDM.

Chr	SNP	EA	OA	PM2.5			GDM		
				BETA	SE	P	BETA	SE	P
10	rs10786736	C	G	0.016	0.004	9.50E-06	-0.038	0.035	0.265
15	rs11855821	A	G	-0.013	0.002	2.10E-07	0.029	0.025	0.234
1	rs12133063	A	C	0.010	0.002	4.00E-06	0.012	0.020	0.563
2	rs13429081	T	A	0.014	0.003	8.90E-07	0.026	0.024	0.282
9	rs1537371	A	C	0.012	0.002	8.50E-09	-0.006	0.020	0.759
10	rs4933236	C	T	0.010	0.002	5.50E-06	-0.023	0.021	0.257
6	rs56068671	T	G	-0.018	0.004	3.60E-06	-0.001	0.027	0.965
3	rs75738250	A	T	0.013	0.003	5.70E-06	0.009	0.024	0.718
8	rs77205736	T	C	0.014	0.002	2.10E-08	-0.042	0.030	0.160
8	rs800507	T	G	0.011	0.003	9.80E-06	0.033	0.023	0.145
17	rs8614	A	C	0.013	0.003	2.80E-06	-0.036	0.028	0.200
6	rs9260734	A	G	0.014	0.003	3.10E-06	-0.073	0.028	0.009
3	rs987204	A	G	0.010	0.002	2.00E-06	0.042	0.021	0.040

SNP, single nucleotide polymorphism; CHR, chromosome; EA, effect allele; OA, other allele; SE, standard error.

Table S3. Characteristics of SNPs used in the MR analysis in the summary statistics reported in the GWAS on PM2.5 and GDM.

Chr	SNP	EA	OA	PM2.5			GDM			F
				BETA	SE	P	BETA	SE	P	
2	rs10203969	T	G	-0.010	0.002	5.50E-06	-0.013	0.023	0.570	20.64
9	rs10961167	A	G	-0.019	0.004	2.00E-06	-0.071	0.047	0.134	22.63
16	rs11075636	G	A	-0.013	0.003	8.90E-06	-0.028	0.028	0.317	19.74
1	rs11209977	G	A	0.014	0.003	8.90E-06	0.007	0.024	0.762	19.74
6	rs114708313	T	A	0.025	0.004	4.20E-08	0.091	0.037	0.013	30.08
12	rs11610713	T	A	0.038	0.008	2.50E-06	0.021	0.070	0.769	22.16
13	rs11616726	G	T	-0.018	0.004	9.00E-06	0.027	0.048	0.582	19.72
4	rs116259394	G	A	-0.035	0.008	8.50E-06	-0.020	0.050	0.683	19.83
8	rs116925111	G	T	0.053	0.011	9.10E-07	0.102	0.076	0.177	24.10
10	rs1172955	A	T	0.011	0.002	5.80E-06	-0.012	0.023	0.601	20.55
22	rs117366154	T	C	-0.019	0.004	6.30E-06	-0.070	0.043	0.097	20.40
22	rs117878224	G	A	-0.048	0.010	1.80E-06	0.133	0.353	0.706	22.78
7	rs1182891	T	G	0.013	0.003	4.40E-06	-0.003	0.028	0.927	21.10
8	rs1217106	G	A	0.013	0.003	1.20E-06	0.027	0.026	0.300	23.58
6	rs12203592	T	C	0.022	0.003	6.20E-17	-0.062	0.061	0.306	69.92
2	rs13035717	T	A	-0.013	0.003	4.60E-06	0.018	0.022	0.419	20.99
4	rs1318845	C	T	-0.014	0.003	2.30E-07	-0.006	0.024	0.818	26.78
9	rs1340247	A	G	-0.010	0.002	7.00E-06	-0.006	0.020	0.755	20.20
5	rs1372504	A	G	0.012	0.002	3.10E-08	0.049	0.021	0.020	30.67
20	rs138685951	T	C	-0.031	0.007	5.60E-06	0.028	0.058	0.628	20.62
12	rs140500108	G	C	-0.022	0.005	5.30E-06	-0.051	0.050	0.306	20.71
12	rs144470316	C	G	-0.051	0.011	9.00E-06	-0.029	0.148	0.842	19.71
9	rs145151841	A	G	0.063	0.014	5.20E-06	0.036	0.084	0.669	20.77
18	rs146171855	C	A	0.092	0.021	8.00E-06	0.078	0.073	0.290	19.95
17	rs146999536	A	C	0.055	0.012	9.40E-06	0.099	0.248	0.690	19.63
17	rs147392452	T	C	-0.028	0.006	8.50E-06	0.006	0.042	0.884	19.82
15	rs151323121	A	G	-0.042	0.009	6.10E-06	0.145	0.135	0.282	20.44
16	rs16957755	C	T	0.022	0.004	7.60E-07	0.004	0.050	0.935	24.46
8	rs17070492	G	C	0.017	0.004	3.00E-06	-0.015	0.034	0.657	21.80
14	rs17103928	A	C	-0.014	0.003	3.70E-06	0.014	0.030	0.642	21.42
18	rs17183854	G	T	-0.043	0.009	4.00E-06	0.012	0.056	0.834	21.25
7	rs17657881	C	T	-0.037	0.008	4.00E-06	-0.019	0.080	0.817	21.27
6	rs1933805	A	G	0.010	0.002	9.40E-06	-0.003	0.020	0.887	19.63

7	rs2091322	C	T	-0.010	0.002	8.80E-06	-0.014	0.022	0.535	19.75
7	rs2141531	G	T	-0.011	0.002	1.30E-06	-0.031	0.023	0.174	23.47
18	rs2278801	G	A	0.010	0.002	6.30E-06	0.026	0.021	0.216	20.39
16	rs2292156	T	G	0.015	0.003	3.40E-07	0.008	0.026	0.765	26.03
6	rs2324898	T	C	0.010	0.002	7.50E-06	0.004	0.021	0.845	20.07
5	rs27152	T	C	-0.011	0.002	3.20E-06	-0.033	0.021	0.121	21.66
11	rs35703574	A	G	-0.025	0.005	3.90E-06	0.088	0.055	0.112	21.31
4	rs45485691	A	G	-0.011	0.002	7.20E-06	-0.018	0.021	0.386	20.15
2	rs4854523	G	A	0.013	0.003	3.40E-06	0.032	0.026	0.213	21.60
1	rs487230	G	A	-0.012	0.003	3.60E-06	0.009	0.024	0.699	21.45
19	rs4926311	T	C	-0.010	0.002	8.20E-06	-0.040	0.021	0.060	19.88
2	rs56181709	T	C	-0.012	0.003	7.10E-06	-0.003	0.022	0.877	20.18
5	rs58824859	C	A	0.012	0.002	1.90E-07	0.021	0.021	0.304	27.14
20	rs6024877	C	T	0.024	0.006	9.70E-06	0.045	0.038	0.243	19.58
20	rs6134106	A	C	0.054	0.012	4.80E-06	0.062	0.048	0.198	20.93
19	rs62119705	T	C	0.011	0.002	8.90E-07	0.034	0.021	0.096	24.16
2	rs62180462	A	G	-0.035	0.008	4.20E-06	0.069	0.113	0.544	21.19
5	rs62368672	C	T	-0.018	0.004	4.00E-06	-0.035	0.051	0.498	21.26
7	rs62484669	A	G	0.018	0.004	8.60E-07	0.047	0.039	0.230	24.21
11	rs628913	T	C	-0.010	0.002	5.40E-06	0.009	0.020	0.640	20.71
2	rs6432776	C	T	0.011	0.002	1.60E-06	-0.015	0.022	0.492	22.97
2	rs6547952	G	T	-0.011	0.002	1.20E-06	0.023	0.021	0.262	23.58
15	rs656813	C	T	-0.014	0.003	8.90E-06	-0.015	0.039	0.707	19.74
3	rs66501886	G	C	-0.010	0.002	9.50E-06	0.031	0.022	0.162	19.60
2	rs6749467	A	G	-0.012	0.002	1.40E-08	-0.016	0.021	0.447	32.23
6	rs6937315	C	T	0.010	0.002	6.20E-06	-0.004	0.022	0.865	20.43
7	rs6960011	G	A	-0.011	0.002	3.70E-06	-0.004	0.021	0.857	21.40
14	rs718059	A	G	-0.014	0.003	4.40E-06	-0.002	0.026	0.941	21.08
18	rs72642437	T	C	0.113	0.019	3.10E-09	0.015	0.041	0.713	35.12
13	rs72655898	G	A	-0.011	0.002	2.40E-06	-0.018	0.022	0.399	22.27
14	rs72700006	C	T	0.020	0.004	2.40E-06	-0.045	0.034	0.183	22.26
2	rs72805044	C	T	-0.037	0.008	7.60E-06	-0.123	0.095	0.192	20.03
5	rs72808024	C	A	-0.016	0.003	9.90E-08	-0.002	0.029	0.934	28.39
1	rs7514956	C	A	-0.013	0.003	2.60E-06	0.016	0.025	0.516	22.12
6	rs75730485	C	T	-0.027	0.006	9.80E-06	0.002	0.052	0.965	19.56
2	rs7573056	C	A	-0.011	0.002	1.50E-07	-0.017	0.020	0.417	27.64
15	rs76230173	A	G	-0.021	0.005	7.60E-06	0.078	0.048	0.104	20.04
12	rs77147615	C	T	0.025	0.006	8.20E-06	-0.035	0.080	0.659	19.88
6	rs77255816	T	C	0.031	0.006	4.20E-08	-0.134	0.105	0.201	30.04
6	rs7750418	G	T	0.010	0.002	6.80E-06	-0.009	0.021	0.659	20.24

6	rs7776279	A	G	0.011	0.002	4.40E-06	0.021	0.023	0.378	21.06
15	rs77962859	C	T	-0.016	0.004	7.30E-06	-0.036	0.033	0.263	20.10
8	rs78230389	G	A	-0.047	0.009	8.10E-07	-0.016	0.246	0.949	24.34
14	rs78467598	G	C	-0.038	0.008	7.60E-07	-0.057	0.086	0.508	24.45
12	rs78539764	C	T	-0.034	0.007	3.70E-07	0.045	0.084	0.595	25.87
20	rs79004040	G	C	-0.032	0.007	8.90E-06	-0.229	0.077	0.003	19.74
10	rs7910200	T	C	-0.010	0.002	6.30E-06	-0.009	0.021	0.679	20.40
3	rs79229868	A	G	0.045	0.010	7.50E-06	0.019	0.042	0.645	20.06
3	rs80123875	T	G	-0.019	0.004	1.30E-06	-0.017	0.037	0.648	23.48
17	rs80347400	G	A	0.014	0.003	6.10E-06	0.009	0.026	0.735	20.46
11	rs875982	T	C	-0.010	0.002	5.70E-06	-0.016	0.020	0.439	20.57
8	rs9644485	C	T	0.010	0.002	4.90E-06	-0.008	0.020	0.702	20.88

SNP, single nucleotide polymorphism; CHR, chromosome; EA, effect allele; OA, other allele; SE, standard error; F, F-statistic .

Table S4. Causal estimates of PM2.5 on GDM in multivariable MR.

Method	Beta	SE	OR	P
Adjusted for BMI	0.861	0.358	2.366(1.664-3.067)	1.607e-02
Adjusted for smoking	0.611	0.182	1.842(1.486-2.199)	8.099e-04
Adjusted for all	0.867	0.360	2.380(1.674-3.085)	1.589e-02

MVMR, multivariable mendelian randomization; BMI, body mass index; SE, standard error; OR, odds ratio;
